# Long non-coding RNA SNHG5 promotes human hepatocellular carcinoma progression by regulating miR-26a-5p/GSK3β signal pathway

**DOI:** 10.1038/s41419-018-0882-5

**Published:** 2018-08-30

**Authors:** Yarui Li, Dan Guo, Yan Zhao, Mudan Ren, Guifang Lu, Yun Wang, Juan Zhang, Chen Mi, Shuixiang He, Xinlan Lu

**Affiliations:** Department of Gastroenterology, the First Affiliated Hospital of Xi’an Jiaotong University, Xi’an, Shaanxi 710061 P.R. China

## Abstract

Accumulating evidence have suggested that long non-coding RNAs (lncRNAs) had malfunctioning roles in the development of human cancers. The present study aimed to investigate the role of lncRNA small nucleolar RNA host gene 5 (SNHG5) in hepatocellular carcinoma (HCC) progression using human tissues and cell lines. The quantitative real-time PCR results showed that SNHG5 was up-regulated in both HCC tissues and hepatoma cell lines and was closely associated with tumor size, hepatitis B virus infection, histologic grade, TNM stage, and portal vein tumor thrombus (PVTT) in HCC patients. Knockdown of SNHG5 induced apoptosis and repressed cell cycle progression, cell growth, and metastasis in hepatoma cell lines, whereas overexpression of SNHG5 had the opposite effects. In vivo functional assay, xenograft tumors grown from SNHG5-knockdown cells had smaller mean volumes than the tumors grown from negative control cells. Further investigations showed that SNHG5 may act as a competing endogenous RNA by competitively binding miR-26a-5p and thereby modulating the derepression of downstream target GSK3β, which were further confirmed by luciferase reporter assay. Functionally, SNHG5 promotes tumor growth and metastasis by activating Wnt/β-catenin pathway and inducing epithelial to mesenchymal transition (EMT). Taken together, SNHG5 promotes HCC progression by competitively binding miR-26a-5p and regulating GSK3β and Wnt/β-catenin signal pathway.

## Introduction

Hepatocellular carcinoma (HCC) is the second leading cause of cancer-related death worldwide^1^. Despite recent advances in the treatment of HCC in surgery, chemotherapy and biologics, it still has a poor prognosis due to tumor metastatic and chemoresistant^2,3^. Tumorigenesis is a complex process involving multiple genetic changes and ultimately leading to the malignant transformation^4^. However, the details of the molecular mechanisms underlying HCC carcinogenesis remain to be elucidated. Therefore, understanding the detailed mechanisms promoting HCC progression will allow for diagnosing and identifying suitable treatment alternatives.

In recent years, emerging evidence suggests that non-coding RNAs (ncRNAs) are involved as important regulators in various physiological and pathological cellular processes^5,6^. Among the large fraction of non-coding transcripts, the class of long non-coding RNAs (lncRNAs), which defined as transcripts longer than 200 nucleotides, is receiving increasing attention and may present new opportunities for disease diagnosis and treatment. In view of tumor biology, dysregulation of lncRNAs could contribute to fundamental aspects of tumor development, and that lncRNAs have more highly diverse roles and are more actively involved in tumorigenesis than previously thought. Emerging studies have pointed to the differential expression patterns of lncRNAs in various tumors and demonstrated their ability to affect cell transformation, tumorigenesis, and metastasis^7^. For instance, H19, HOTAIR, MALAT1, TUG1, GAS5, and CCAT1, several well-studied lncRNAs, have been reported to play significant roles in cancer initiation and development^8–13^. Although thousands of lncRNAs have been identified and extensive gene expression and variation analyses have linked their alteration to fundamental cancer progression, there were still many interesting questions need careful consideration, including how lncRNAs are deregulated in cancer, what their role is in tumorigenesis and what underlying mechanisms drive these relationships.

Small nucleolar RNA host gene 5 (SNHG5), one of the well-defined cytoplasmic lncRNAs, also called U50HG, is 524 bp in length. SNHG5 is composed of six exons and two snoRNAs, U50 and U50’, which are encoded in introns 4 and 5, respectively^14^. Aberrant expression of SNHG5 has been reported in several human cancers including malignant melanoma, colorectal cancer, and gastric cancer^15–18^. As far as we know, the functional role of SNHG5 in HCC is completely unknown.

In the present study, we aimed to identify and investigate the role of cytoplasmic lncRNA SNHG5 in HCC tumorigenesis. We found that SNHG5 was up-regulated in HCC tissues and in hepatoma cell lines. Knockout of SNHG5 inhibits the malignant biological characteristics of HCC cells. Although we have learned that many lncRNAs function in the tumor cells, little is known about the mechanism of action of lncRNAs. Recently, competing endogenous RNAs (ceRNAs) emerged as a new concept, which means lncRNAs act as molecular sponges for microRNAs hence relieving repression of their target mRNAs^19–21^. By bioinformatics analysis and follow-up experimental verification, we found that SNHG5 acts as a ceRNA by competitively binding miR-26a-5p thus impairing its repression on target gene GSK3β. Additionally, SNHG5 play an oncogenic role in liver tumorigenesis by activating the Wnt/β-catenin signal pathway and leading to epithelial-mesenchymal transition (EMT). Hence, we here assessed the expression pattern of SNHG5 RNA and provided new insights into its significance and biological role in promoting HCC survival.

## Results

### SNHG5 is upregulated in HCC and correlated with poor progression

**Fig. 1:**
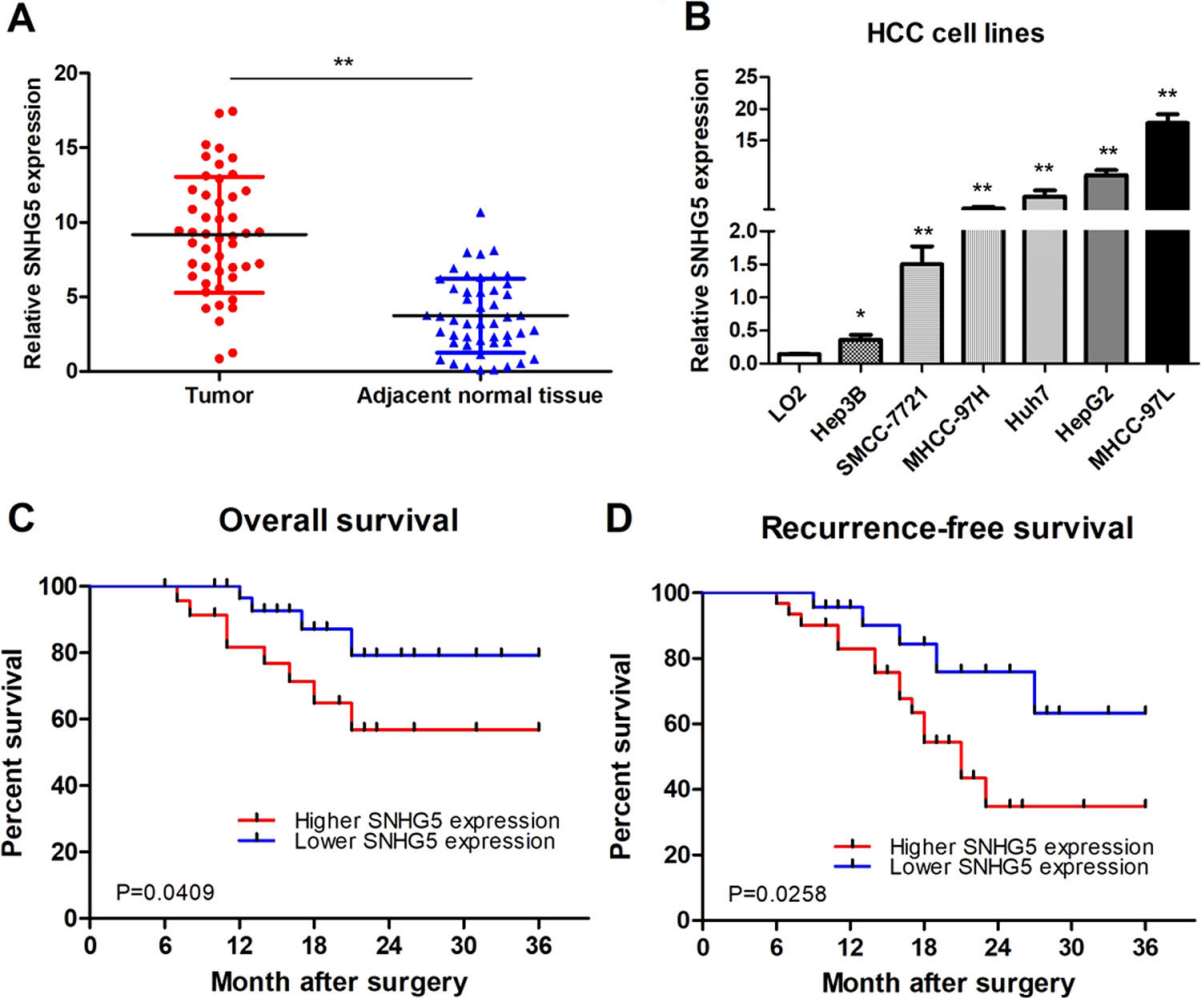
SNHG5 was overexpressed in HCC tissues and hepatoma cell lines. **a** qRT-PCR analysis of SNHG5 expression in 48 patients with HCC. Data were shown as the means ± s.d. *P < 0.05; **P < 0.01. **b**–**d** qRT-PCR analysis of SNHG5 expression in HCC tissues according tumor sizes (<5 cm and ≥5 cm), TNM stage (III/IV and I/II), and PVTT(Yes or No), **P < 0.01. **e** qRT-PCR analysis of SNHG5 expression in HCC cell lines and the immortalized human hepatic cell line LO2, *P < 0.05; **P < 0.01. **f**, **g** Kaplan–Meier analysis of OS and RFS based on SNHG5 expression levels in HCC patients. The median level of SNHG5 is used as the cutoff. Patients with HCC are divided into a high-expression group and a low-expression group

Expression of SNHG5 was analyzed by qRT-PCR in 48 HCC and matched adjacent non-malignant tissues. Results showed that SNHG5 expression was significantly higher in HCC tissues compared to non-malignant tissues (Fig. 1a). In addition, SNHG5 expression is higher in the HCC cell lines compared with the LO2 (immortalized, normal human hepatic cell line) (Fig. 1b). Results from clinical studies indicated that aberrant expression of SNHG5 was closely associated with the clinicopathological parameters of HCC, such as Tumor size, HBV infection, histologic grade, TNM stage, and portal vein tumor thrombus (PVTT) (Table 1). Overall survival (OS) was defined as the interval between resection and death or the last follow-up visit. Recurrence-free survival (RFS) was defined as the interval between treatment and the first diagnosis of metastasis or recurrence. Kaplan–Meier and log-rank analyses suggested that patients with high SNHG5 expression have shorter OS and higher recurrence rates than those with low SNHG5 expression (Fig. 1c, d). The multivariate analysis indicated that SNHG5 expression was an independent predictor of OS and RFS (Table 2). These results demonstrated that overexpresssion of SNHG5 played a key role in the development and progression of HCC.

**Table 1:** Relationship between SNHG5 expression and clinical characteristics of HCC patients

Clinical factors	No.of cases	SNHG5 expression		*P* value
		Low (*n* = 22)	High (*n* = 26)	
Age (years)				
<59	20	12	8	0.096
≥59	28	10	18	
Gender				
Male	38	16	22	0.478
Female	10	6	4	
Tumor size				
<5 cm	23	15	8	0.010^*^
≥5;cm	25	7	18	
HBV infection				
Positive	37	13	24	0.006^*^
Negative	11	9	2	
AFP (μg/L)				
<400	18	8	10	0.881
≥400	30	14	16	
Histologic grade				
Well and moderate	28	16	12	0.005^*^
Low	20	6	14	
TNM stage				
I/II	32	19	13	0.008^*^
III/IV	16	3	13	
PVTT				
Yes	13	2	11	0.010^*^
No	35	20	15	

*PVTT* portal vein tumor thrombus

*The *P* values with significance are marked in bold

**Table 2:** Univariate and multivariate analysis of clinical factors by Cox proportional hazard regression model for overall survival and recurrence-free survival in HCC patients

Parameter	Overall survival						Recurrence-free survival					
	Univariate analysis			Multivariate analysis			Univariate analysis			Multivariate analysis		
	HR	*P* value	95% CI	HR	*P* value	95% CI	HR	*P* value	95% CI	HR	*P* value	95% CI
Age	0.845	0.701	0.358–1.993				0.912	0.822	0.408–2.039			
Gender	0.561	0.320	0.180–1.752				0.475	0.121	0.185–1.216			
Tumor size	6.441	0.003^*^	1.878–12.092	2.418	0.251	0.536–10.915	3.137	0.017^*^	1.230–7.997	0.839	0.783	0.241–2.924
Serum AFP (mg/L)	1.177	0.731	0.463–2.990				1.001	0.998	0.429–2.339			
HBV infection	1.192	0.707	0.478–2.975				0.996	0.993	0.410–2.418			
Histologic grade	6.348	0.000^*^	2.429–12.591	0.375	0.229	0.076–1.856	4.161	0.010^*^	1.403–12.336	0.502	0.328	0.127–1.993
TNM stage	5.787	0.000^*^	2.226–15.046	5.854	0.002^*^	1.905–17.990	4.452	0.001^*^	1.890–10.490	3.900	0.006^*^	1.490–10.240
PVTT	5.003	0.011^*^	1.450–7.263	4.191	0.031^*^	1.139–11.416	6.040	0.000^*^	2.474–14.742	6.032	0.008^*^	1.586–12.942
SNHG5 expression	4.033	0.013^*^	1.340–11.325	4.74	0.015^*^	1.350–6.640	3.548	0.013^*^	1.311–9.602	3.690	0.020^*^	1.229–11.082

*The *P* values with significance are marked in bold

### Downregulation of SNHG5 induces apoptosis and represses cell cycle progression, proliferation, and metastasis of HCC cells in vitro

**Fig. 2:**
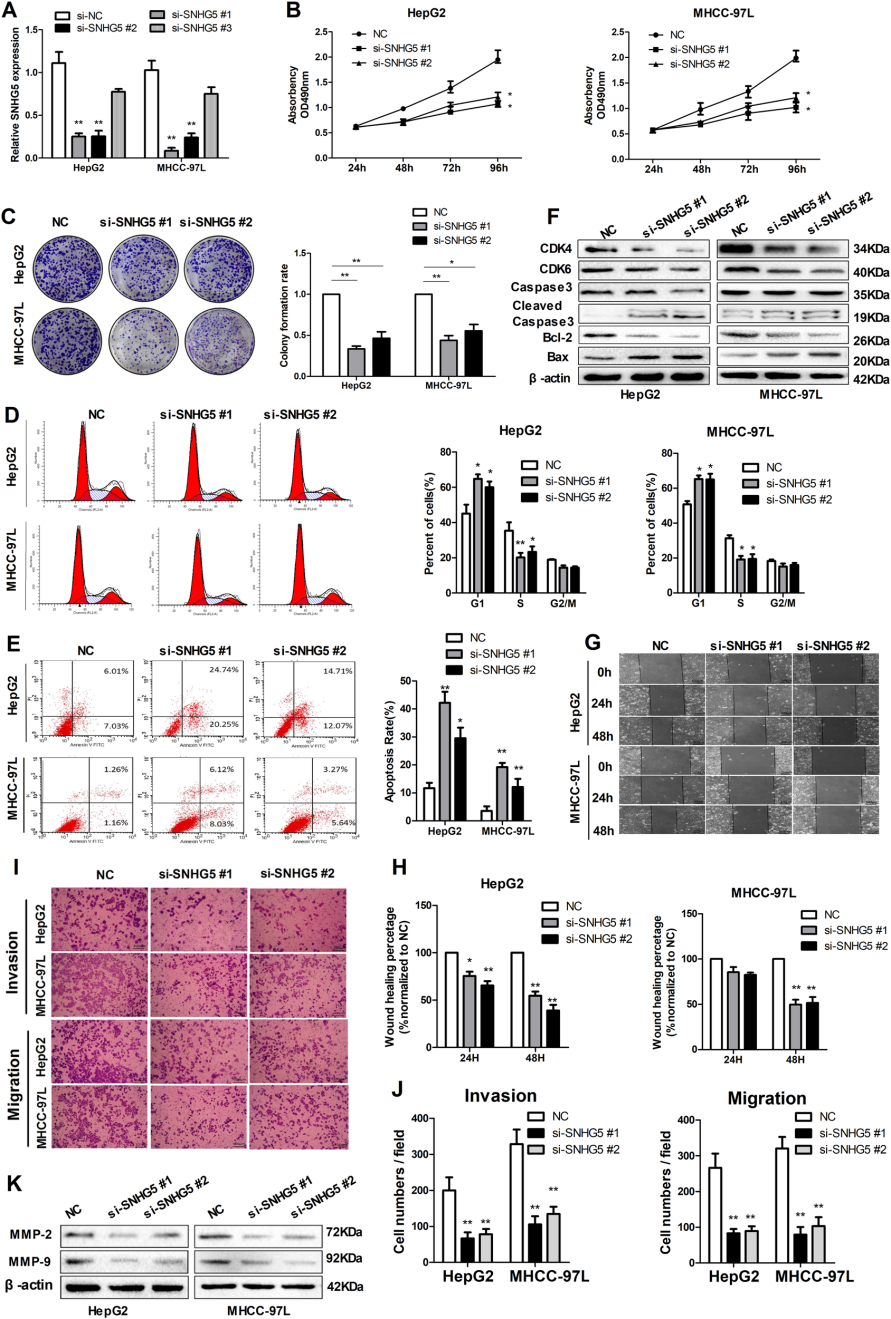
Downregulation of SNHG5 induces apoptosis and promotes proliferation, migration and invasion in HCC lines. Data are shown as the means ± s.d. *P < 0.05; **P < 0.01. **a** qRT-PCR analysis of SNHG5 expression following transfected HCC cells with three different SNHG5-siRNAs, **P < 0.01. **b** MTT assays showing that silencing of SNHG5 inhibited the proliferation of HCC cells. *P < 0.05. **c** SNHG5 knockdown caused a decrease in the clonogenic survival of HCC cells. *P < 0.05; **P < 0.01. **d**, **e** FACS analysis showed downregulation of SNHG5 induces apoptosis and represses cell cycle progression in HCC cells. *P* < 0.05; **P < 0.01. **f** Common cell cycle-related and apoptosis-related proteins expression levels detected by western blot analysis following SNHG5 silencing. **g**, **h** Representative images of wound healing assays after SNHG5 silencing. *P < 0.05; **P < 0.01. **i**, **j** Transwell assays showed SNHG5 knockdown reduced the migration and invasion of HCC cells. (scale bars = 50 mm). **P < 0.01. **k** Expression levels of MMP-2 and MMP-9 after SNHG5 knockdown were analyzed by western blot

To investigate the potential biological function of SNHG5,3 specific siRNA (siRNA #1-#3) against SNHG5 gene transcript were introduced into HepG2 and MHCC-97L cells, which have higher endogenous SNHG5 expression levels. siRNA#1 and siRNA #2 produced the greatest reduction in endogenous SNHG5 expression (Fig. 2a), therefore, we chose these two siRNAs for the following experiments. Firstly, growth curves measured by MTT assay showed that downregulation of SNHG5 significantly decreased HCC cell growth (Fig. 2b), and colony formation assay verify this results by demonstrated that SNHG5 knockdown caused a decrease in the clonogenic survival of HepG2 and MHCC-97L cells compared with negative control (NC) (Fig. 2c). Secondly, the flow cytometric analysis (FACS) revealed the differences of cell cycle distribution in SNHG5 downregulation. A significant increase in the proportion of cells in G1 and a decrease in the proportion of cells in S phase were found when SNHG5 knockdown in HepG2 and MHCC-97L cells (Fig. 2d). Consistent with the above data, the expression of G1/S-phase checkpoint proteins CDK4 and CDK6 markedly decreased when SNHG5 was downregulated (Fig. 2f). In short, these data strongly indicate that SNHG5 regulates tumorigenesis by facilitating cell cycle progression. In addition, FACS analysis showed that the percentage of apoptotic cells (HepG2 and MHCC-97L) significantly increased when SNHG5 was downregulated (Fig. 2e). Consistent with the FACS data, the expression levels of apoptosis-related protein Bax and cleaved caspase3 markedly increased and Bcl-2 decreased when SNHG5 was downregulated (Fig. 2f). In short, these data strongly indicate that SNHG5 influences tumorigenesis by promoting cell proliferation and facilitating cell cycle progression.

As described before, higher expression of SNHG5 was found in advanced TNM stages and PVTT patients. Thus, we speculated that SNHG5 might be critical for tumor metastasis. To validate this hypothesis, two approaches were used to analyze differences in cell invasion and migration following SNHG5 silencing. Firstly, wound healing assay used on assess motility of cells at different time points, and the results suggested that downregulation of SNHG5 suppressed cell migration in HepG2 and MHCC-97L cells (Fig. 2g, h). Consistent with the wound healing assay results, Transwell assays showed downregulation of SNHG5 reduced the migration and invasion cells compared with the NC (Fig. 2i, j). In addition, SNHG5 knockdown inhibited the expression of two matrix metalloproteinases, MMP-2 and MMP-9, that are closely related to metastasis (Fig. 2k). In general, these data indicate that SNHG5 plays a crucial role in promoting cell metastasis.

### Overexpression of SNHG5 represses cell apoptosis, induces cell cycle progression, and promotes proliferation, invasion, and migration in vitro

**Fig. 3:**
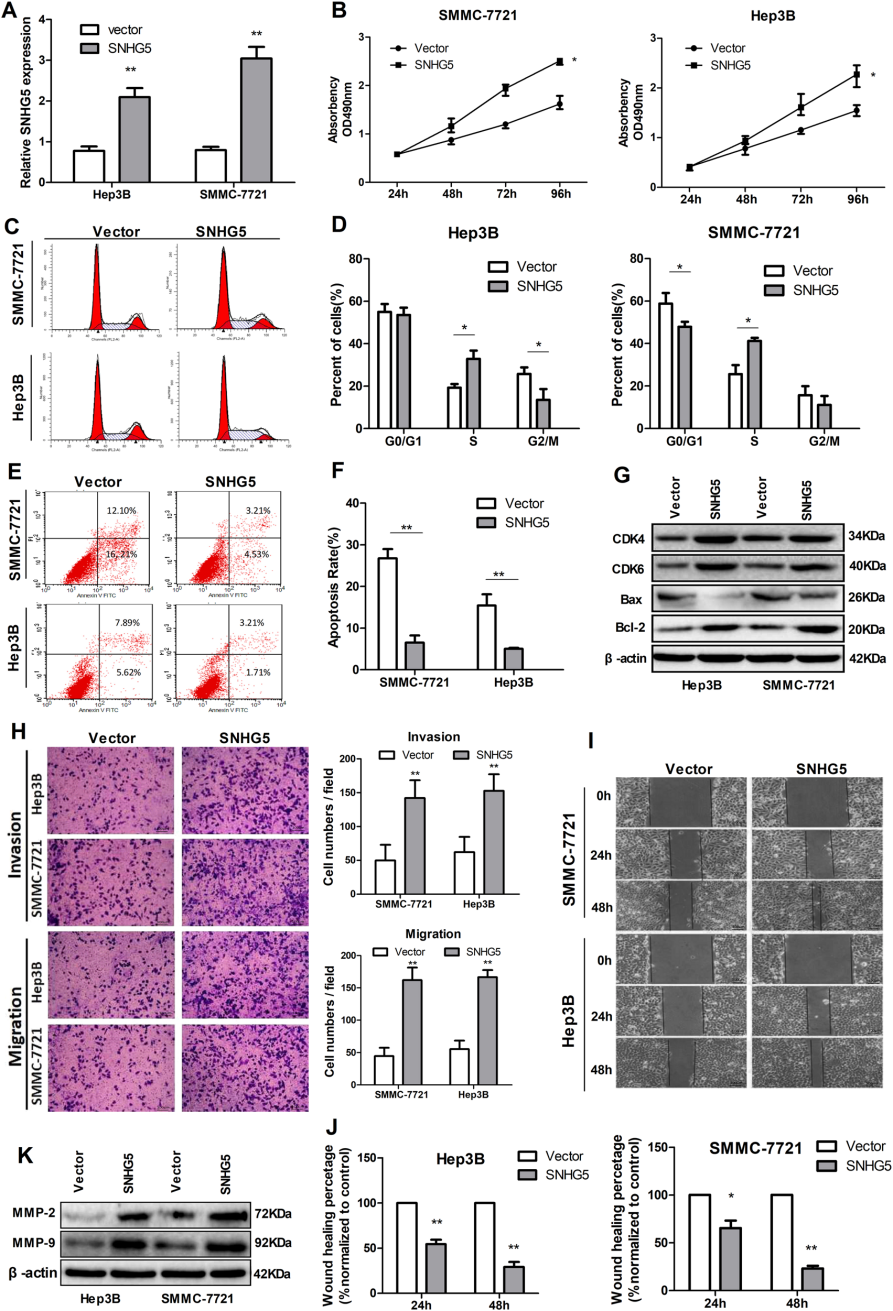
Overexpression of SNHG5 represses cell apoptosis and promotes hepatoma cell proliferation, invasion and migration in vitro. **a** qRT–PCR analysis of SNHG5 expression after SNHG5 overexpression. **P < 0.01. **b** MTT assay showed SNHG5 overexpression promotes HCC cells proliferation. *P < 0.05. **c**–**f** FACS analysis showed overexpression of SNHG5 represses cell apoptosis, induces cell cycle progression. **g** Common cell cycle-related and apoptosis-related proteins expression levels detected by western blot analysis following SNHG5 upregulation. **h**–**j** Transwell and wound healing assays after SNHG5 overexpression. *P < 0.05; **P < 0.01. **k** Expression levels of MMP-2 and MMP-9 in HCC cells after SNHG5 overexpression were analyzed by western blot

We further examined the role of SNHG5 by assessing the effect of its overexpression in SMCC-7721 and Hep3B cells, which have lower endogenous SNHG5 levels. A pCMV-SNHG5 expression plasmid was conducted for cell experiment. SNHG5 expression in SMCC-7721 and Hep3B cells dramatically increased after transfection with the pCMV-SNHG5 vector compared with an empty vector (Fig. 3a). Growth curves produced by MTT assay showed that SNHG5 upregulation significantly increased SMCC-7721 and Hep3B cell growth (Fig. 3b). FACS analysis showed that overexpression of SNHG5 promoted cell cycle progression (Fig. 3c, d) and represses cell apoptosis in SMCC-7721 and Hep3B cells (Fig. 3e, f). The expression of G1/S-phase checkpoint proteins CDK4, CDK6 and the expression levels of apoptosis-related protein Bcl-2 markedly increased when SNHG5 was overexpressed, but the Bax expression increased (Fig. 3g). Meanwhile, wound healing and transwell assays were used to analyze differences in cell invasion and migration following SNHG5 overexpression. As the results showed, SNHG5 upregulation significantly promoted the invasion and migration of SMCC-7721 and Hep3B cells compared with the control (Fig. 3h–j). Consistent with the functional tests, SNHG5 overexpression promoted the expression of two matrix metalloproteinases, MMP-2 and MMP-9, which are closely related to metastasis (Fig. 3k). Overall, SNHG5 exerts an oncogenic effect in hepatoma cells.

### Knockdown of SNHG5 represses tumor growth and metastasis in vivo

**Fig. 4:**
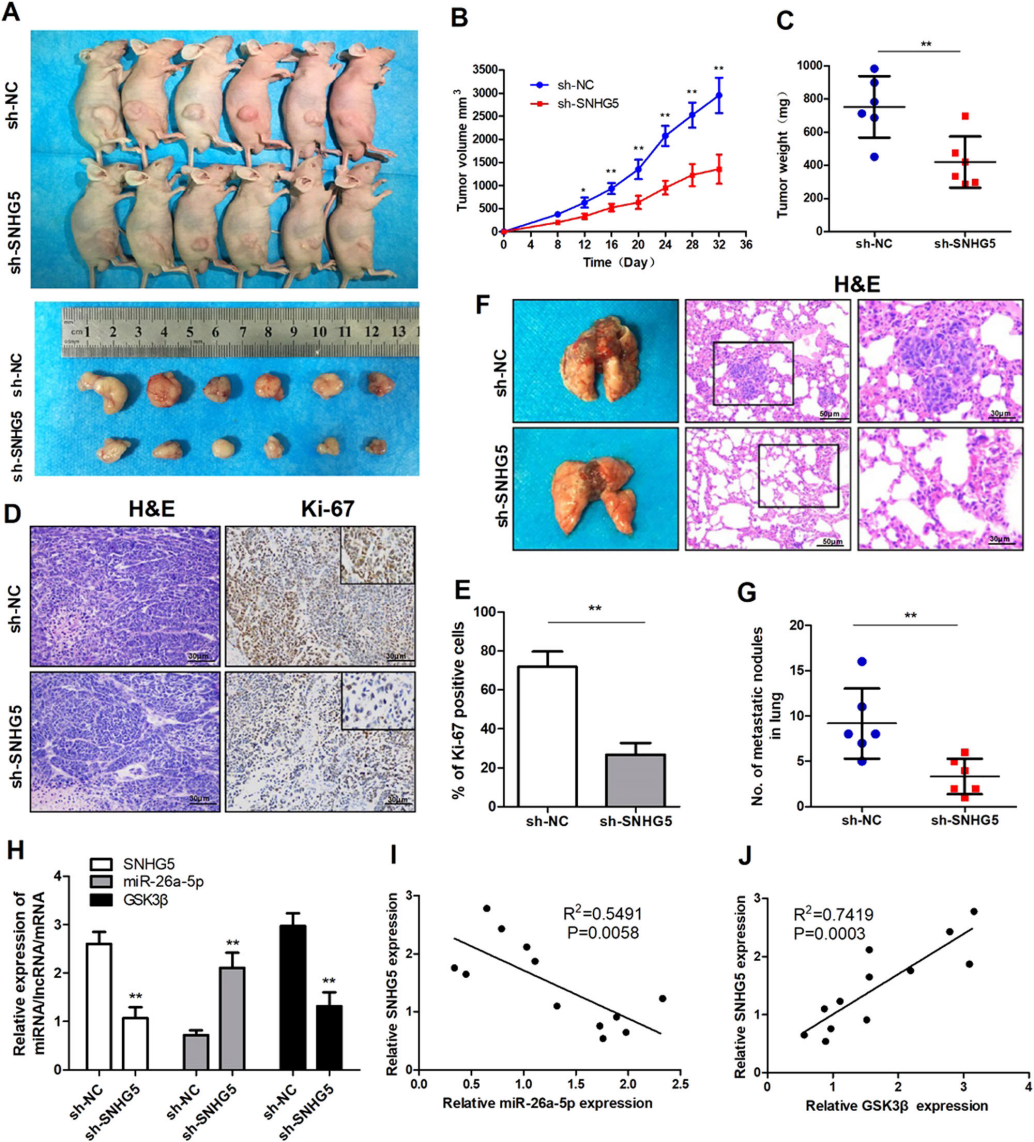
Knockdown of SNHG5 represses tumor growth and metastasis in vivo. **a** Representative images of nude mice modles and formed tumors that were subcutaneously injected with SNHG5-shRNA and NC-shRNA cells. **b**, **c** Effect of SNHG5 knockdown on HCC growth in vivo according to the tumor growth curve and tumor weight. *P < 0.05; **P < 0.01. **d**, **e** Representative images of HE and IHC staining patterns for Ki-67 in tumor xenografts of nude mice (200×). **P < 0.01. **f**, **g** Representative images of pulmonary metastatic models and HE staining of metastatic nodules in the lungs. (200× and 400×), **P < 0.01. **h** The expression of SNHG5, miR-26a-5p, and GSK3β in xenograft tumors were detected by qRT-PCR. **P < 0.01. **i**, **j** Pearson’s correlation analysis of the relationship between SNHG5 and miR-26a-5p, SNHG5, and GSK3β expression levels in xenograft tumors

HepG2 cells stably transfected with SNHG5-shRNA and NC were subcutaneously injected in male nude mice for 5 weeks. We found that SNHG5-shRNA dramatically inhibited tumor growth compared to the NC group (Fig. 4a). The tumor growth curve and tumor weight suggested that SNHG5 knockdown effectively suppressed tumor growth compared with the NC group (Fig. 4b, c). Additionally, SNHG5 inhibition dramatically reduced Ki-67 expression compared with the NC group (Fig. 4d, e). Next, we evaluated the effects of SNHG5 on HCC tumor metastasis using pulmonary nude mouse model. There were clearly a greater number of pulmonary metastatic nodules in the lung in the NC group compared with the SNHG5-knockdown group. Furthermore, HE staining of lung sections showed that SNHG5 knockdown reduced the number and size of visible lung metastases (Fig. 4f, g). Other than, the expression of SNHG5, miR-26a-5p, and GSK3β were detected in xenograft tumors. The result of qRT-PCR showed that SNHG5 and GSK3β expression were decreased in SNHG5-shRNA xenograft tumors, but the miR-26-5p expression were increased (Fig. 4h). The further Pearson’s correlation analysis suggested that miR-26a-5p expression was inversely correlated with SNHG5 and positively correlated with GSK3β expression in xenograft tumors (Fig. 4i, j).

### SNHG5 acts as a competing ceRNA and regulates GSK3β expression by competitively binding miR-26a-5p

**Fig. 5:**
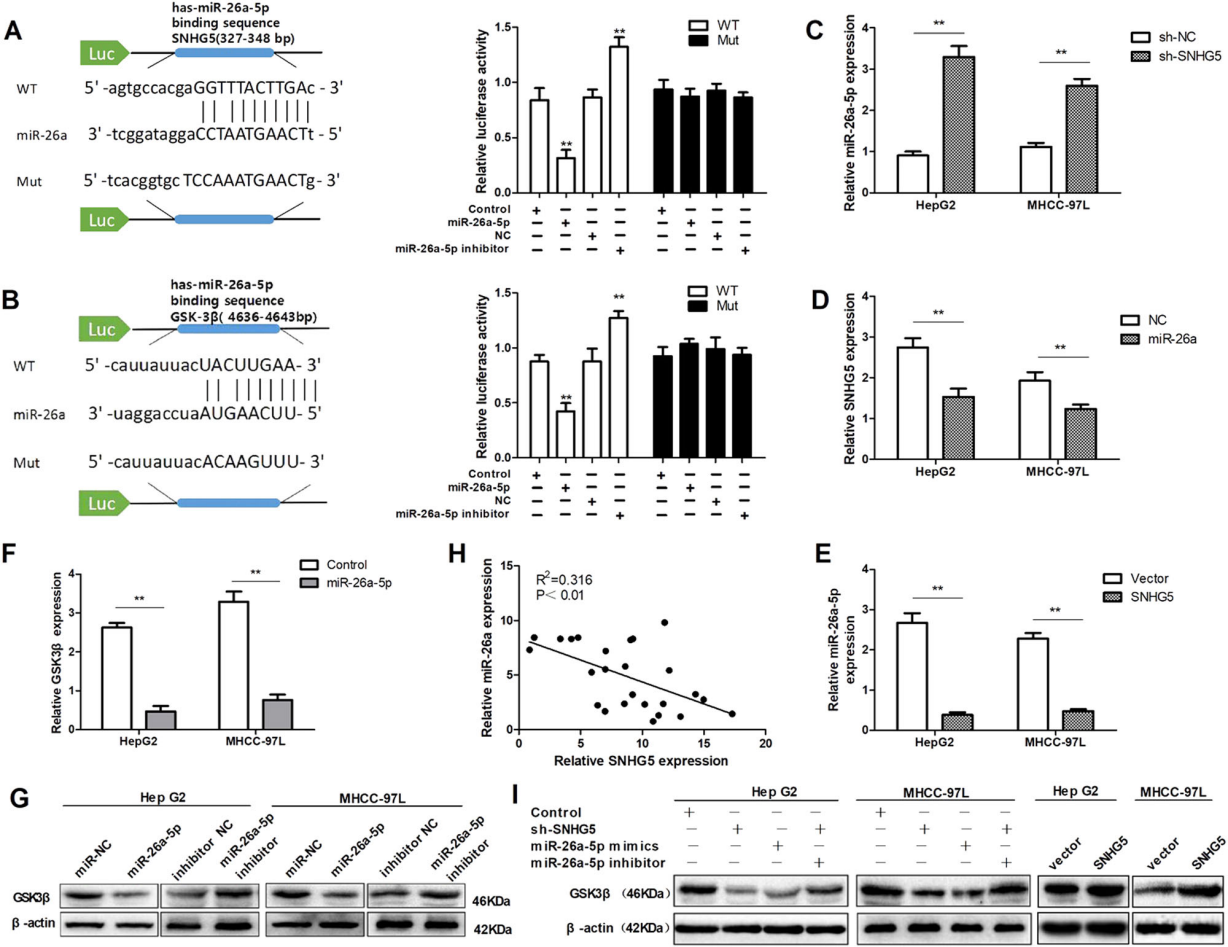
SNHG5 acts as a competing endogenous RNA and regulates GSK3β expression by competitively binding miR-26a-5p. **a** Luciferase reporter assay was applied to verify the targeted binding effect between SNHG5 3′UTR and miR-26a-5p. **P < 0.01. **b** Luciferase reporter assay was applied to verify the targeted binding effect between GSK3β 3′UTR and miR-26a-5p. **P < 0.01. **c**–**e** qRT-PCR analysis of SNHG5 and miR-26a-5p expression following transfected HepG2 and MHCC-97L cells with different regents. *P < 0.05, **P < 0.01. **f** The GSK3β mRNA levels after miR-26a-5p overexpression were deceted by qRT-PCR. **P < 0.01. **g** The GSK3β protein levels after miR-26a-5p overexpression or downregulation were deceted by western blot. **h** Pearson’s correlation analysis of the relationship between SNHG5 and miR-26a-5p expression levels in HCC tissues. **i** Western blot analyses of GSK3β expression after knockdown or upregulated SNHG5, while the inhibition of miR-26a-5p reversed the change in GSK3β expression

Recent studies have shown that many lncRNAs act as competing ceRNAs by competitively binding miRNAs. To investigate the mechanism underlying the role of SNHG5 in HCC tumorigenesis, we investigated whether miRNAs are involved in HCC progression. We used starBase^22^ and DIANA LncBase^23^ software for biological information prediction. As shown in Fig. 5a, SNHG5 contains a potential binding site for miR-26a-5p. Co-transfection of 293T cells with pCMV-SNHG5-WT vector and miR-26a-5p mimics significantly reduced luciferase reporter activity compared with cells transfected with pCMV-SNHG5-Mut, and downregulation of miR-26a by miR-26a-5p inhibitor increased the luciferase activity of SNHG5. However, mutating this putative binding site of miR-26a-5p resulted in compete abrogation of above effects.

Since miRNAs are known to target specific genes to regulate tumor progression, to identify potential target genes of miR-26a-5p, we searched for candidate genes using TargetScan Human 7.1 software^24^. Bioinformatics analysis predicted that miR-26a-5p contains a potential binding site for the GSK3β 3′-UTR. Several papers have reported that GSK3β is a target of miR-26a-5p^25,26^. GSK3β is also considered an important transcription factor involved in the Wnt signal pathway, which is associated with tumor growth and metastasis. We next constructed luciferase reporter vectors containing the 3′-UTR of GSK3β, and luciferase reporter assay result showed co-transfection cells with pCMV-GSK3β-WT vector and miR-26a-5p mimics significantly inhibited luciferase reporter activity, but miR-26a-5p inhibitor increased the luciferase activity. However, pCMV-GSK3β-Mut in miR-26a-5p’s putative targeting sites didn’t resulted in these effects (Fig. 5b). Meanwhile, we found that knockdown of SNHG5 could increase miR-26a-5p expression and upregulation of SNHG5 suppressed the expression of miR-26a-5p (Fig. 5c, d). Conversely, overexpression of miR-26a-5p suppressed the expression of SNHG5 in HepG2 and MHCC-97L cells (Fig. 5e). In addition, increased expression of miR-26a-5p markedly suppressed GSK3β RNA and protein levels in HepG2 and MHCC-97L cells. These results were reversed when miR-26a-5p was inhibited (Fig. 5f, g). Pearson’s correlation analysis suggested that miR-26a-5p expression was inversely correlated with SNHG5 in HCC tissues (Fig. 5h). Furthermore, western blot analysis showed that knockdown of SNHG5 or overexpression of miR-26a-5p decreased GSK3β expression levels in HepG2 and MHCC-97L cells. As a rescue experiment, inhibition of miR-26a-5p expression in SNHG5-knockdown cells reversed the decrease in GSK3β expression (Fig. 5i). These results suggested that SNHG5 regulates GSK3β expression by sequestering endogenous miR-26a-5p. Taken together, we provided evidence that SNHG5 acts as an endogenous “sponge” by binding miR-26a-5p and thus abolishing miRNA-induced repression of GSK3β.

### SNHG5 promotes HCC cell growth by inhibiting miR-26a-5p/GSK-3β axis

**Fig. 6:**
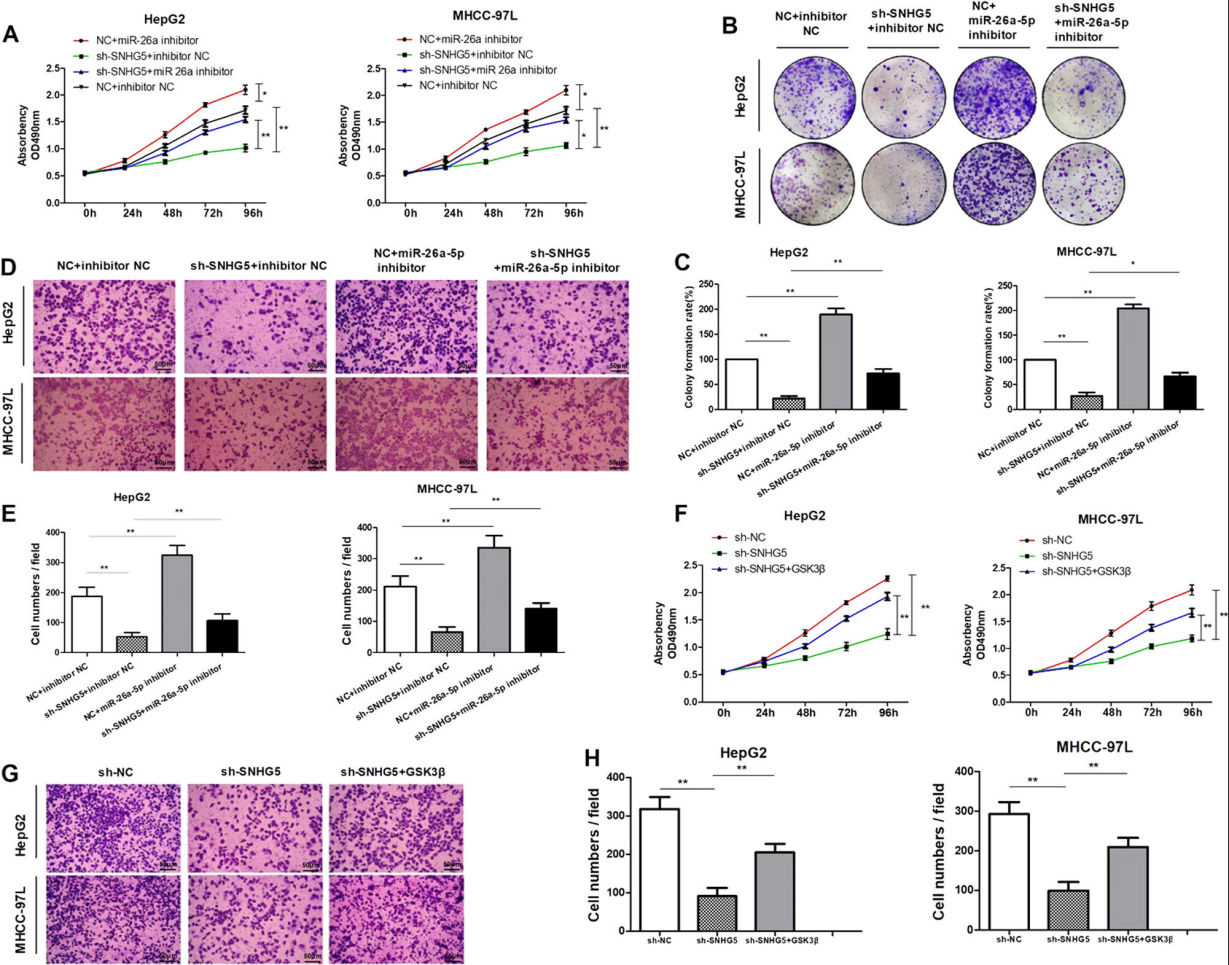
SNHG5 promotes HCC cell growth by inhibiting miR-26a-5p/GSK-3β axis. **a**–**c** MTT and colony formation assays demonstrated SNHG5 reversed the growth inhibitory role of miR-26a-5p in HepG2 and MHCC-97L cells. *P < 0.05,**P < 0.01. **d**, **e** Transwell invasion assays demonstrated SNHG5 reversed the inhibitory role of miR-26a-5p on invasion in HepG2 and MHCC-97L cells. **P < 0.01. **f** MTT assays demonstrated GSK3β reversed the growth inhibitory role of SNHG5-shRNA in HepG2 and MHCC-97L cells.**P < 0.01. **f** Transwell invasion assays demonstrated GSK3β reversed the inhibitory role of SNHG5-shRNA on migration in HepG2 and MHCC-97L cells. **P < 0.01

Our previous study indicated that miR-26a-5p functions as tumor suppressor in HCC^27^. Since that we found SNHG5 directly binds to miR-26a-5p, we next investigated the regulation of HCC cell growth by SNHG5 in combination with miR-26a-5p. MTT assay was used to determine SNHG5-shRNA HCC cells growth in response to miR-26a-5p inhibitor transfection. The results showed cell proliferation of HepG2 and MHCC-97L cell lines was reduced when SNHG5 knockdown compared with NC and inhibitor NC group, cell growth was promoted when miR-26a-5p inhibition, however, the inhibitory effect of SNHG5-shRNA on HCC cell growth could be partially restored by miR-26a-5p inhibitor (Fig. 6a). Consistent with the MTT assay results, colony formation(Fig. 6b, c) and Transwell assay (Fig. 6d, e) indicated that the inhibitory effect of SNHG5-shRNA on HCC cell growth and invasion could be partially restored by miR-26a-5p inhibition. After then, we investigated the regulation of HCC cell growth by SNHG5 and GSK3β. Results showed that SNHG5-shRNA suppressed cell growth and invasion, however, these effects could be partially restored by GSK3β overregulation (Fig. 6f-h). Overall, these results suggested that SNHG5 regulates HCC cell growth and metastasis by inhibiting miR-26a-5p/GSK-3β axis.

### SNHG5 induced EMT by competitively binding miR-26a-5p and activating Wnt/β-catenin pathway

**Fig. 7:**
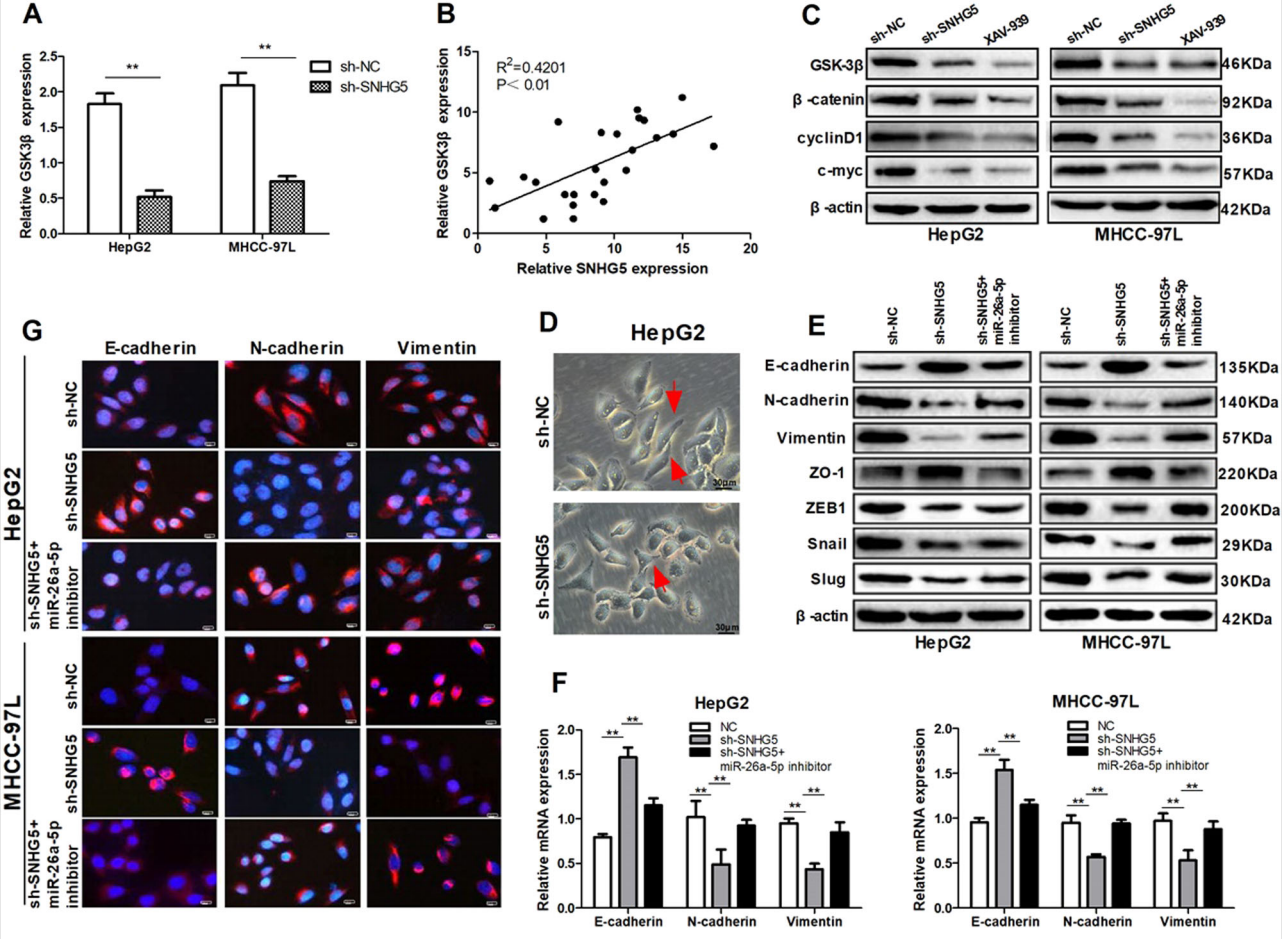
SNHG5 induced EMT by competitively binding miR-26a-5p and activating Wnt/β-catenin pathway. **a** qRT–PCR analysis of GSK3β mRNA expression after SNHG5 knockdown. **P < 0.01. **b** Pearson’s correlation analysis of the relationship between SNHG5 and GSK3β mRNA expression levels in HCC tissues. **c** Western blot analysis of the key components of Wnt/β-catenin pathway protein expression following SNHG5 knockdown or co-transfection with shRNA and miR-26a-5p inhibitor. **d** HepG2 cells adopted EMT-related cell morphology in response to SNHG5 depletion. **e**, **f** Expression levels of EMT markers in HCC cells after transfected with shRNA- SNHG5 or co-transfection with shRNA and miR-26a-5p inhibitor were analyzed by western blot and quantitative RT-PCR. *P < 0.05; **P < 0.01. **g** Expression levels of EMT markers in HCC cells after SNHG5 knockdown or co-transfection with shRNA and miR-26a-5p inhibitor were analyzed by immunofluorescence

It has been revealed that GSK3β is one of the key components in Wnt/β-catenin pathway. Hence, we speculated that SNHG5 promote HCC progression by upregulating GSK3β and activating the Wnt/β-catenin pathway. Results showed that knockdown of SNHG5 significantly reduced the expression of GSK3β mRNA (Fig. 7a), and Pearson’s correlation analysis showed GSK3β mRNA expression was significantly positively correlated with SNHG5 expression levels in HCC tissues (Fig. 7b). Furthermore, western blot analysis indicated knockdown of SNHG5 significantly decreased the protein expression levels of the crucial components of Wnt/β-catenin pathway in HepG2 and MHCC-97L cells, which had a similar effect as Wnt/β-catenin pathway inhibitor XAV-939 (Fig. 7c).

**Fig. 8:**
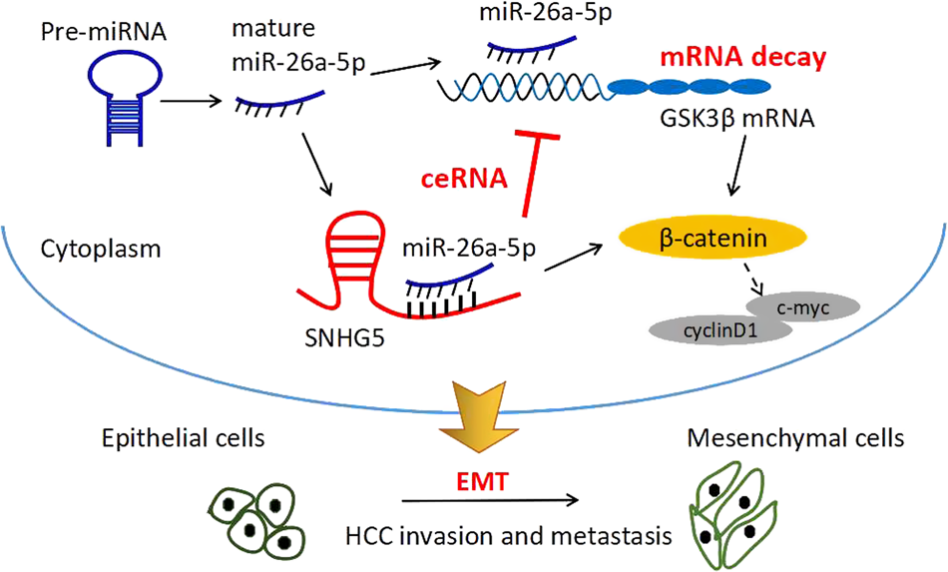
A schematic model depicting the functions of SNHG5 during the invasion and metastasis of HCC. SNHG5 induced EMT by competitively binding miR-26a-5p and regulating GSK3β and Wnt/ β-catenin signal pathway

Previous studies suggested that Wnt/β-catenin pathway play important role in tumor EMT, hence we speculated that SNHG5 induces EMT by activates Wnt/β-catenin pathway. Firstly, we examined the effect of SNHG5 on cell phenotype, knockdown of SNHG5 induced the reversion of mesenchymal like morphological feature into an epithelial phenotype in HepG2 cells (Fig. 7d).The rescue experiments was conducted to prove that SNHG5 promote tumor EMT by competitively binding miR-26-5p. QRT-PCR and western blot analysis showed that SNHG5-knockdown increased epithelial markers E-cadherin, ZO-1 and decreased mesenchymal markers N-cadherin and vimentin, however, these effects could be partially restored by miR-26a-5p inhibitor (Fig. 7e, f). Consistent with western blot analysis, rescue experiment of IF also showed that SNHG5-shRNA resulted in increased expression of E-cadherin but decreased expression of N-cadherin and vimentin compared to that in NC cells, and miR-26a-5p inhibitor restored the effect (Fig. 7g). All these results suggested that SNHG5 induced EMT potentially by competitively binding miR-26a-5p and activating Wnt/β-catenin pathway (Fig. 8).

## Discussion

Recently, emerging researches have revealed the functional roles of lncRNAs in a variety of human tumors^28,29^, which provided insights into the molecular mechanisms of tumorigenesis. SNHG5 belongs to a large family of non-coding multiple small nucleolar RNA host gene and most often residing in the introns of the host genes. Several studies confirmed that SNHG5 played oncogenic or suppressive role in various cancers. However, the mechanisms of SNHG5 in HCC have not been thoroughly elaborated. In the present study, we found that SNHG5 was overexpressed in HCC tissues and potentially correlated with HCC formation and progression.

In this study, we confirmed that SNHG5 expression in HCC tissue was significantly higher than adjacent normal liver tissues. Specifically, SNHG5 expression was remarkably higher in larger tumors and more advance stages of tumor development. The strong correction between SNHG5 expression levels and HCC prognosis or therapeutic outcome was shown in our study. Kaplan–Meier survival analysis demonstrated a correction of higher SNHG5 expression in tumors with poor survival than lower SNHG5 expression. Multivariate Cox regression analysis suggested SNHG5 expression was independent from other clinical covariates, suggesting that it could be a useful prognostic biomarker to help identify high-risk HCC patients. Taken together, SNHG5 acts as an oncogene in hepatocarcinogenesis and can be considered as a potential prognostic indicator for HCC. To further explore the mechanism, the SNHG5 function was investigated using siRNA or sh-RNA mediated knockdown experiments. Knockdown of SNHG5 significantly decreased HCC cell growth, cell cycle progression, migration and invasion, and inhibited cell apoptosis in vitro. Meanwhile, xenograft tumor model showed knockdown of SNHG5 inhibited tumor growth and metastasis in vivo. Collectively, our observations demonstrated SNHG5 plays a key role in HCC initial, development, and progression.

Although we have demonstrated that SNHG5 act as an oncogene in HCC, the potential mechanism by which SNHG5 participates in tumorigenesis remains to be elucidated. Recently, a novel regulatory mechanism has been identified that lncRNAs can act as endogenous “sponges” by binding to miRNAs and regulating their function^30^. That is, lncRNAs inhibit subsequent miRNA binding to target mRNAs by acting as competitive ceRNAs^31,32^. In this study, we aimed to discover the potential molecular mechanisms by which SNHG5 functions as a ceRNA in HCC progression. Bioinformatics analysis (starBase^22^ and DIANA LncBase^23^ software) showed that SNHG5 contains a potential binding site for miR-26a-5p. In addition, our previous study showed that miR-26a-5p was downregulated in HCC and inhibited proliferation and metastasis of HCC cells^27^. Therefore, we speculated that SNHG5 may mediate HCC cell process by interacting with miR-26a-5p, and through luciferase reporter and qRT-PCR assay, we found that SNHG5 directly binds to miR-26a-5p and there was an interactive suppression between them. Subsequently, bioinformatics analysis showed that GSK3β was a direct target of miR-26a-5p, and several researches have confirmed this correction^25,26^. GSK3β is also considered an important transcription factor involved in the Wnt signal pathway, which is associated with tumor growth and tumorigenicity^33^. Our result showed SNHG5 could regulate GSK3β expression by sequestering endogenous miR-26a-5p. These results suggested that SNHG5 acts as an endogenous “sponge” by binding miR-26a-5p and thus abolishing miRNA-induced repression of GSK3β.

Increasing evidence identified that tumor migration and invasion is tightly involved in EMT, which by induced the tumor cells transforming from polar epithelial-like cells to mesenchymal phenotype cells^34,35^. Previous studies indicated that Wnt/β-catenin pathway play important role in tumor EMT^36,37^, and many lncRNAs have been demonstrated to modulated cancer cell function by targeting the key components of the Wnt/β-catenin pathway. For instance, lncRNA MEG3 promotes the proliferation of glioma cells through targeting Wnt/β-catenin signal pathway;^38^ lncRNA PLIN2 promotes the development of chronic myelogenous leukemia via the GSK3 and Wnt/β-catenin signaling pathways^39^. In our study, we demonstrated that SNHG5 regulated the Wnt/β-catenin signaling pathway, and knockdown of SNHG5 increased epithelial markers E-cadherin and ZO-1 but decreased mesenchymal related markers vimentin, N-cadherin, snail, and ZEB1. These results suggested that knockdown of SNHG5 suppresses cell EMT via modulating Wnt/β-catenin pathway.

In summary, our study illustrated that highly expressed SNHG5 was a novel oncogene which promoted the tumorigenesis and progression of HCC through competitively binding miR-26a-5p. Furthermore, SNHG5 induced tumor EMT by activating the Wnt/β-catenin pathway. This study provided a mechanistic understanding of the oncogenic role of SNHG5 HCC and indicated that SNHG5 might be an important prognostic factor and a potential therapeutic target in HCC.

## Materials and methods

### Clinical specimens and cell lines

HCC specimens and the matched adjacent non-malignant tissues (3–5 cm distal to the edge of tumor) were collected from patients with HCC being treated at the First Affiliated Hospital of Xi’an Jiaotong University after obtaining informed consent. None of patients received any chemotherapy or radiotherapy treatments before surgery. HCC diagnosis was confirmed by histopathology. The consents used in the study were reviewed and approved by the the Hospital’s Ethics Committee. The human HCC cell lines (Hep3B, HepG2, SMCC-7721, MHCC-97L, MHCC-97H, Huh7 and the immortalized human hepatic cell line LO2 were obtained from the Type Culture Collection of the Chinese Academy of Sciences (Shanghai, China).All cells are epithelial-like morphology, growth of adherent. All cells were stored in liquid nitrogen and cultured in DMEM (Gibco, USA) supplemented with 10% fetal bovine serum (FBS, Gibco, USA), 100 μg/mL streptomycin and 100 U/mL penicillin (Sigma, USA). All cells were maintained at 37 °C in 5% CO^2^ humidified incubator.

### RNA isolation, and quantitative real-time PCR

Total RNA from specimens and cultured cells was extracted using Trizol reagent (Invitrogen, Carlsbad, CA) according to the manufacturer’s protocol. Then the cDNA was obtained according to the protocol of PrimeScript^TM^ RT Master Mix Kit (Takara, Japan) and Mir-X miRNA qRT-PCR SYBR Kit (Takara, Japan). Quantitative real-time PCR (qRT-PCR) was performed using SYBR Premix Ex Taq™II (Takara)on Thermal Cycler CFX6 System (Bio-Rad). U6and β-actin were used as endogenous controls, respectively. The relative gene expression level was calculated using the 2^−ΔΔCt^ method. The primers used in this study were presented in Supplementary Table 1

### Plasmid constructions and cell transfection assay

The small interfering RNA (siRNA) against SNHG5 and NC were designed by Genepharma (Shanghai, China). The siRNA sequences were presented in Supplementary Table 1. To construct stable cell lines, a shRNAs were designed and inserted into the GV248 vector by Genechem (Shanghai, China). Stable clones with SNHG5-shRNA were selected for 4 weeks using puromycin. MiRNA mimics and inhibitors (for miR-26a-5p) were purchased from RiboBio (Guangzhou, China). SNHG5 and GSK3β were cloned into the expression vector pCMV (Invitrogen) for overexpression. pCMV-SNHG5-MUT and pCMV-GSK3β-MUT (GeneCopoeia, China) vector containing mutations at the putative miR-26a-5p binding site were generated by site-directed mutagenesis. Transfection assay was done using Lipofectamine 2000 (Invitrogen, CA, USA) according to the manufacturer’s protocol at approximately 50–70% cell confluence.

### Colony formation assay

Cells were transfected with siRNA SNHG5 or siRNA NC, pCMV/SNHG5 or empty vector as described above. Twenty-four hours after routine incubation, transfected cells were trypsinized, centrifuged, counted and replated at a density of 500 cells/6 cm plate. After 12 days, individual cells grew into colonies containing at least 50 cells, the cell colonies were fixed with 3.7% methanol, stained with 0.1% crystal violet and counted.

### Cell viability assay (MTT assay)

MTT (3-(4, 5-dimethylthiazol-2-yl)-2, 5-diphenyltetra-zolium bromide) assay was used to detect cell proliferation. HepG2 and MHCC-97L cells were seeded in 96-well plates at a density of 3–5 × 10^4^ cells/well and then transfected with the indicated reagent. After transfection for 24, 48, 72, and 96 h, cells were subsequently cultured in medium with 0.5 mg/ml MTT and kept in the dark for 4 h. Following removed the supernatant and added 150 μl DMSO. Cell viability was determined by measuring the optical density (OD) at 490 nm by EnSpire Multimode Plate Reader (PerkinElmer).

### Flow cytometry

For apoptosis analyses, HepG2 and MHCC-97L cells transfected with appropriate reagent were trypsinized and resuspended in 1 × binding buffer at 1 × 10^6^cells/mL. Taken 100 μL cell suspension, 5 μL of FITC-Annexin V and 5 μL propridium iodide (PI) were added and incubated in the dark for 15 min. The reaction was terminated with the addition of 400 μL 1 × binding buffer and analyzed with FACS Calibur (Becton Dickinson). For cell cycle analyses, HepG2 and MHCC-97L cells were harvested after transfection for 48 h, washing with phosphate-buffered saline (PBS)and fixed in 75% ethanol at 4 °C overnight. RNA was removed from the preparations by incubating the cells with RNase A (Sigma) at 37 °C for 30 min. Cells were then stained with PI solution for 30 min at room temperature and analyzed on FACS Calibur (Becton Dickinson).

### Western blot analysis

After transfection for 48 h, proteins were collected from cells using RIPA Buffer (Pierce) containing proteinase and phosphatase inhibitors. Each protein sample was electrophoresed with SDS-polyacrylamide gel electrophoresis (4% stacking and 10% SDS–PAGE separating gels), transferred to the PVDF membrane (Merck Millipore). The membrane was blocked with 5% non-fat milk and incubated with corresponding primary antibodies (Supplementary Table 2) at 4 °C overnight. Then washed the membrane extensively and followed by incubation with secondary antibodies (Zhuangzhi Biology, China) at room temperature for 1 h. The results were subsequently subjected to immunoblotting analysis using the ECL immunoblotting kit (Millipore, USA) according to the manufacturer’s protocol. Image J software-based analysis was used to quantify the bands obtained through Western blot (Supplementary figures).

### Transwell and wound healing assay

Transwell assay was performed to assessed cell migration and invasion after transfection for 48 h. To measure cell invasion, the upper chambers of 8 μm pore size insert (Merck Millipore) were pre-coated with Matrigel (BD Biosciences). 5 × 10^4^ cells in serum-free medium were seeded into the top chambers, DMEM medium containing 10% FBS was added to the lower chamber. For migration, Matrigel was not needed to coat the upper membrane, then the same invasion assay was performed. After incubation for 24 h at 37 °C, the chambers were fixed using 4% paraformaldehyde, stained with crystal violet solution for 30 min, and washed three times with PBS. Stained cells were observed under optical microscope,counted and calculated the mean. Cell migration was also assessed using wound healing assay. HCC cells (1 × 10^6^ cells/well) were treated with the indicated reagents, and wounds were made using a 100 μl plastic pipette tip. After after 24 h and 48 h of wound formation, the wound size was measured and photographed.

### Immunofluorescence (IF)

Cells were cultured on glass coverslips for 24 h to confluence, then fixed in 4% paraformaldehyde at room temperature for 15 min. After washing with PBS, the adherent cells were permeabilized using 0.5% Triton X-100 and blocked for1h with 10% goat serum in 1% BSA/PBS. Then the cells incubated with primary antibody 4 °C overnight and secondary antibodies with a appropriate dilution (Supplementary Table 2) according to the manufacturer’s protocol. After three washes with PBS, coverslips were counter stained with DAPI and imaged with a invert fluorescent microscope (Nikon Eclipse Ti-S).

### Luciferase reporter assay

Bioinformatics tools were used to analyze the miR-26a-5p binding sites on SNHG5 and GSK3β binding sites on miR-26a-5p. Cells were cultured in 96-well plates and proliferated to 60–80% confluence after 24 h before transfection. Plasmid vector including the wild-type (WT) or mutant (Mut) 3′UTR of SNHG5 or GSK3β (GeneCopoeia, China) together with miR-26a-5p mimics or NC (RiboBio) were co-transfected using the Lipofectamine 2000 reagent (Invitrogen, Carlsbad, CA). After transfection for 48 h, the Dual Luciferase Assay Kit was conducted to examine the luciferase activity according to the manufacturer’s instructions. Renilla luciferase activity was used as control.

### Tumor formation in BALB/c nude mice

Male athymic 4-week-old BALB/c nude mice (purchase from the Central Laboratory of Animal Science, Xi’an jiaotong University, China) were randomly divided into two groups and received 5 × 10^6^/200 μl HepG2 cells stably transfected with SNHG5-shRNA or NC-shRNA via subcutaneously injected. Tumor sizes were measured every 4 days, and the mice were sacrificed 5 weeks after injection. For lung metastasis models, HepG2 cells stably transfected with SNHG5-shRNA or NC-shRNA were suspended at 5 × 10^6^ cells/mL. Then, 100 μl suspended cells were injected into the tail vein of nude mice (4 weeks old). Six weeks later, the mice were sacrificed, the lungs were removed and fixed with formaldehyde solution. Visible tumors on the lung surfaces were counted and hematoxylin-eosin staining (H&E) were performed to evaluate the lung metastases.

### H&E staining and Immunohistochemistry (IHC)

For H&E staining, tissues were fixed in 10% neutral buffered formalin for 24 h. Then, embedded in paraffin wax and cut 4 mm thick, deparaffinized with xylene, and processed with agraded ethanol series. Sections were stained with H&E and observed using microscope. For IHC, paraffin-embedded tissues were cut into 4 mm sections. The sections were deparaffinized, rehydrated, and stained with primary antibodies with a appropriate dilution (Supplementary Table 2) at 4 °C overnight. The slides were treated with secondary antibody and then treated with DAB. Finally, the slides were counter stained with hematoxylin and visualized under the light microscope.

### Statistical analysis

Data are presented as means ± SD and analyzed by SPSS statistics 20.0 (SPSS, Chicago, USA) and Graphpad Prism 5.0 (CA, USA). Student’s *t*-test, *χ*2 test, and Fisher’s exact tests were was performed to compare the differences of the two groups. Correlation between two groups was analyzed using Pearson’s correlation coefficient analysis. The Kaplane-Meier test was used to estimate the OS and RFS and multivariate Cox regression analysis was used to test for independent prognostic factors. A value of *P* < 0.05 was considered significant.

## Electronic supplementary material

Supplementary figures

Supplementary Tables

## References

[1] Global battle against cancer won’t be won with treatment alone–effective prevention measures urgently needed to prevent cancer crisis. Central Eur. J. Publ. Health **22**, 23–28 (2014). 24844101

[2] Torre LA (2015). Global cancer statistics, 2012. CA Cancer J. Clin..

[3] Ashhab AA, Rodin H, Debes JD (2017). Hepatocellular carcinoma diagnosis and surveillance: socioeconomic factors don’t seem to matter, unless you are an immigrant-Authors-reply. J. Hepatol..

[4] El-Serag HB, Rudolph KL (2007). Hepatocellular carcinoma: epidemiology and molecular carcinogenesis. Gastroenterology.

[5] Kondo Y, Shinjo K, Katsushima K1 (2017). Long non-coding RNAs as an epigenetic regulator in human cancers. Cancer Sci..

[6] Chen X, Fan S, Song E (2016). Noncoding RNAs: new players in cancers. Adv. Exp. Med. Biol..

[7] Schmitt AM, Chang HY (2016). Long noncoding RNAs in cancer pathways. Cancer Cell.

[8] Li X (2017). The role of long noncoding RNA H19 in gender disparity of cholestatic liver injury in multidrug resistance 2 gene knockout mice. Hepatology.

[9] Heubach J (2015). The long noncoding RNA HOTAIR has tissue and cell type-dependent effects on HOX gene expression and phenotype of urothelial cancer cells. Mol. Cancer.

[10] Malakar P (2017). Long noncoding RNA MALAT1 promotes hepatocellular carcinoma development by SRSF1 upregulation and mTOR activation. Cancer Res..

[11] Lin PC (2016). Long noncoding RNA TUG1 is downregulated in non-small cell lung cancer and can regulate CELF1 on binding to PRC2. BMC Cancer.

[12] Ye K (2017). Long noncoding RNA GAS5 suppresses cell growth and epithelial-mesenchymal transition in osteosarcoma by regulating the miR-221/ARHI pathway. J. Cell. Biochem..

[13] Deng L, Yang SB, Xu FF, Zhang JH (2015). Long noncoding RNA CCAT1 promotes hepatocellular carcinoma progression by functioning as let-7 sponge. J. Exp. Clin. Cancer Res..

[14] Damas ND (2016). SNHG5 promotes colorectal cancer cell survival by counteracting STAU1-mediated mRNA destabilization. Nat. Commun..

[15] Zhao L (2016). Long non-coding RNA SNHG5 suppresses gastric cancer progression by trapping MTA2 in the cytosol. Oncogene.

[16] Lee J, Ryu J, Lee C (2016). Strong cis-acting expression quantitative trait loci for the genes encoding SNHG5 and PEX6. Medicine.

[17] Zhao L (2017). The lncRNA SNHG5/miR-32 axis regulates gastric cancer cell proliferation and migration by targeting KLF4. FASEB J..

[18] Ichigozaki Y (2016). Serum long non-coding RNA, snoRNA host gene 5 level as a new tumor marker of malignant melanoma. Exp. Dermatol..

[19] Cesana M (2011). A long noncoding RNA controls muscle differentiation by functioning as a competing endogenous RNA. Cell.

[20] Salmena L, Poliseno L, Tay Y, Kats L, Pandolfi PP (2011). A ceRNA hypothesis: the Rosetta Stone of a hidden RNA language?. Cell.

[21] Liu T (2017). Curcumin suppresses proliferation and in vitro invasion of human prostate cancer stem cells by ceRNA effect of miR-145 and lncRNA-ROR. Gene.

[22] Li JH, Liu S, Zhou H, Qu LH, Yang JH (2014). starBasev2.0: decoding miRNA-ceRNA, miRNA-ncRNA and protein-RNA interaction networks from large-scale CLIP-Seq data. Nucleic Acids Res..

[23] Paraskevopoulou MD (2013). DIANA-LncBase: experimentally verified and computationally predicted microRNA targets on long non-coding RNAs. Nucleic Acids Res..

[24] Agarwal V, Bell GW, Nam JW, Bartel DP (2015). Predicting effective microRNA target sites in mammalian mRNAs. eLife.

[25] Lin G (2017). MiR-26a enhances invasive capacity by suppressing GSK3β in human lung cancer cells. Exp. Cell Res..

[26] Suh JH (2012). Up-regulation of miR-26a promotes apoptosis of hypoxic rat neonatal cardiomyocytes by repressing GSK-3β protein expression. Biochem. Biophys. Res. Commun..

[27] Li Y (2017). MicroRNA-26a inhibits proliferation and metastasis of human hepatocellular carcinoma by regulating DNMT3B-MEG3 axis. Oncol. Rep..

[28] Misawa A, Takayama KI, Inoue S (2017). Long non-coding RNAs and prostate cancer. Cancer Sci..

[29] Bhan, A., Soleimani, M. & Mandal, S. S. Long noncoding RNA and cancer: a new paradigm. Cancer Res. **77**, 3965–3981 (2017). 10.1158/0008-5472.CAN-16-2634 PMC8330958 28701486

[30] Tay Y, Rinn J, Pandolfi PP (2014). The multilayered complexity of ceRNA crosstalk and competition. Nature.

[31] Tan JY, Marques AC (2016). Marques, miRNA-mediated crosstalk between transcripts: the missing “linc”?. Bioessays.

[32] Song C (2017). The global view of mRNA-related ceRNA cross-talks across cardiovascular diseases. Sci. Rep..

[33] Liu XF, Li XY, Zheng PS, Yang WT (2018). DAX1 promotes cervical cancer cell growth and tumorigenicity through activation of Wnt/β-catenin pathway via GSK3β. Cell Death Dis..

[34] Lo UG, Lee CF, Lee MS, Hsieh JT (2017). The role and mechanism of epithelial-to-mesenchymal transition in prostate cancer progression. Int. J. Mol. Sci..

[35] Dominguez, C., David, J. M. & Palena, C. Epithelial-mesenchymal transition and inflammation at the site of the primary tumor. Semin Cancer Biol, **47**, 177–184 2017. Aug 18. [Epub ahead of print]. 10.1016/j.semcancer.2017.08.002 PMC5698091 28823497

[36] Ma F (2017). MiR-23a promotes TGF-beta1-induced EMT and tumor metastasis in breast cancer cells by directly targeting CDH1 and activating Wnt/β-catenin signaling. Oncotarget.

[37] Wang S (2017). Fibulin-3 promotes osteosarcoma invasion and metastasis by inducing epithelial to mesenchymal transition and activating the Wnt/β-catenin signaling pathway. Sci. Rep..

[38] Gong X, Huang M (2017). Long non-coding RNA MEG3 promotes the proliferation of glioma cells through targeting Wnt/β-catenin signal pathway. Cancer Gene Ther..

[39] Sun, C. et al. CEBPA-mediated upregulation of the lncRNA PLIN2 promotes the development of chronic myelogenous leukemia via the GSK3 and Wnt/β-catenin signaling pathways. Am J Cancer Res. **7**, 1054–1067(2017). PMC5446474 28560057

