# Supplementary material for: Long non-coding RNA SNHG5 promotes human hepatocellular carcinoma progression by regulating miR-26a-5p/GSK3β signal pathway
Source: Cell Death Dis. 2018 Aug 30;9(9):888. doi: 10.1038/s41419-018-0882-5 (PMC6117363; doi:10.1038/s41419-018-0882-5)
Supplement: Supplementary file 1 — Supplementary figures [file 41419_2018_882_MOESM1_ESM.docx]

**Supplementary figures**

**Western blot quantitative statistics**

**Figure2F**

**
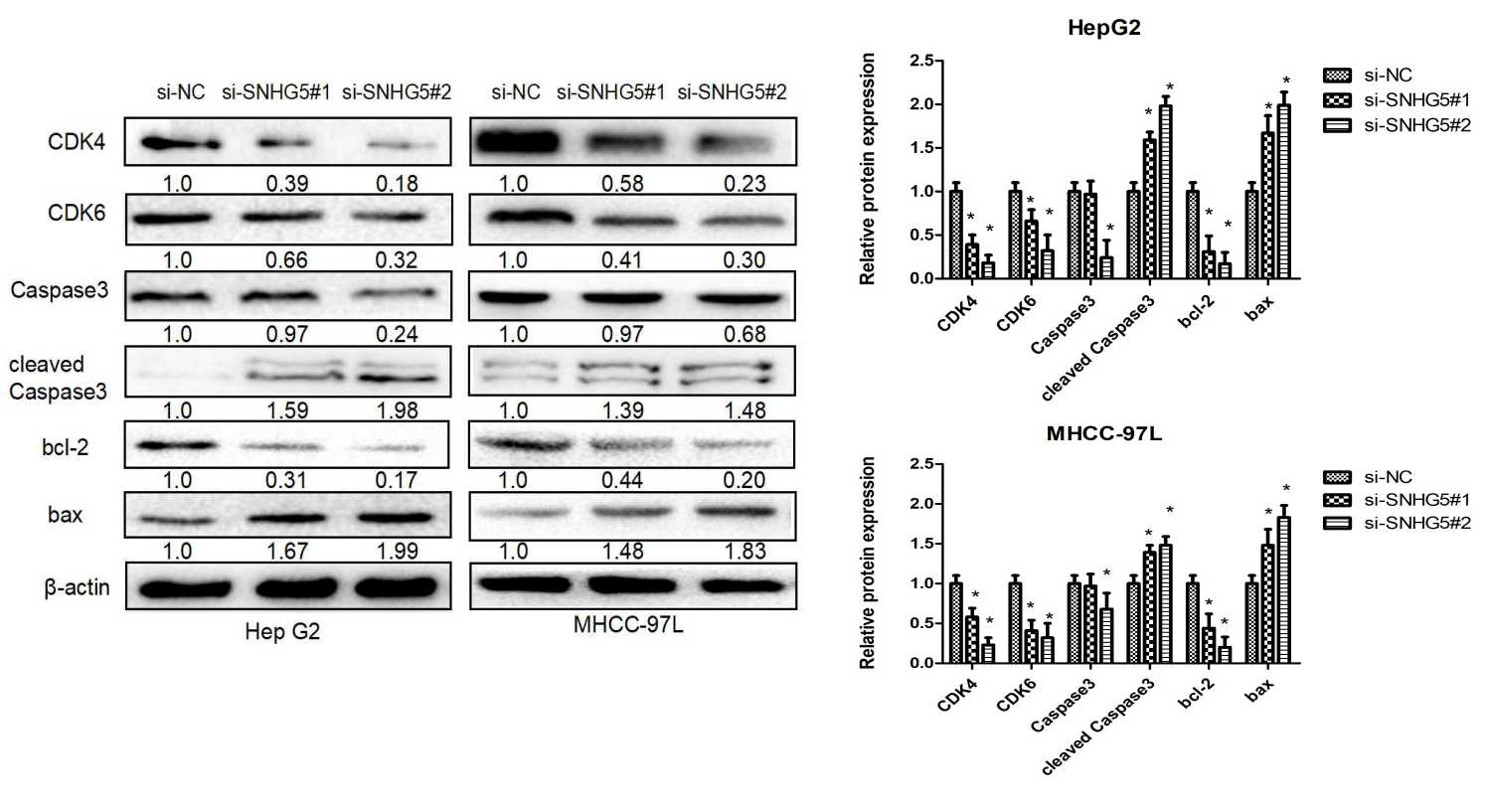
**

Fig.2F. Common cell cycle-related and apoptosis-related proteins expression levels detected by western blot analysis following SNHG5 silencing. Quantitative analysis of protein relative expression by Image J.

**Figure2K**

**
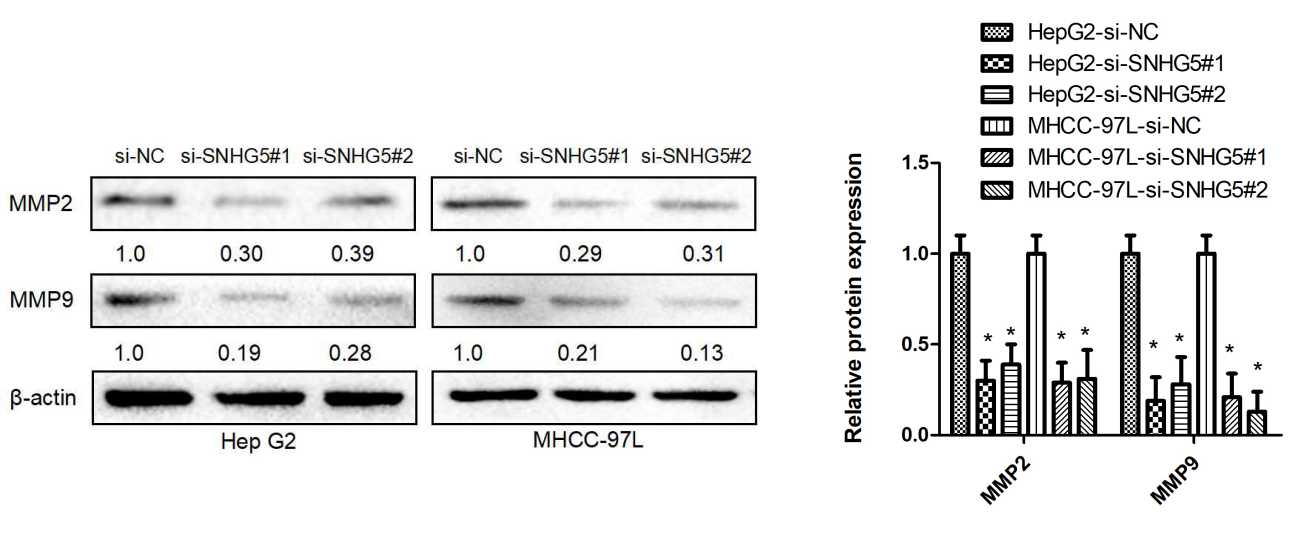
**

Fig.2K. The MMP-2 and MMP-9 expression after SNHG5 knockdown were analyzed by western blot.Quantitative analysis of protein relative expression by Image J.

**Figure3G**

**
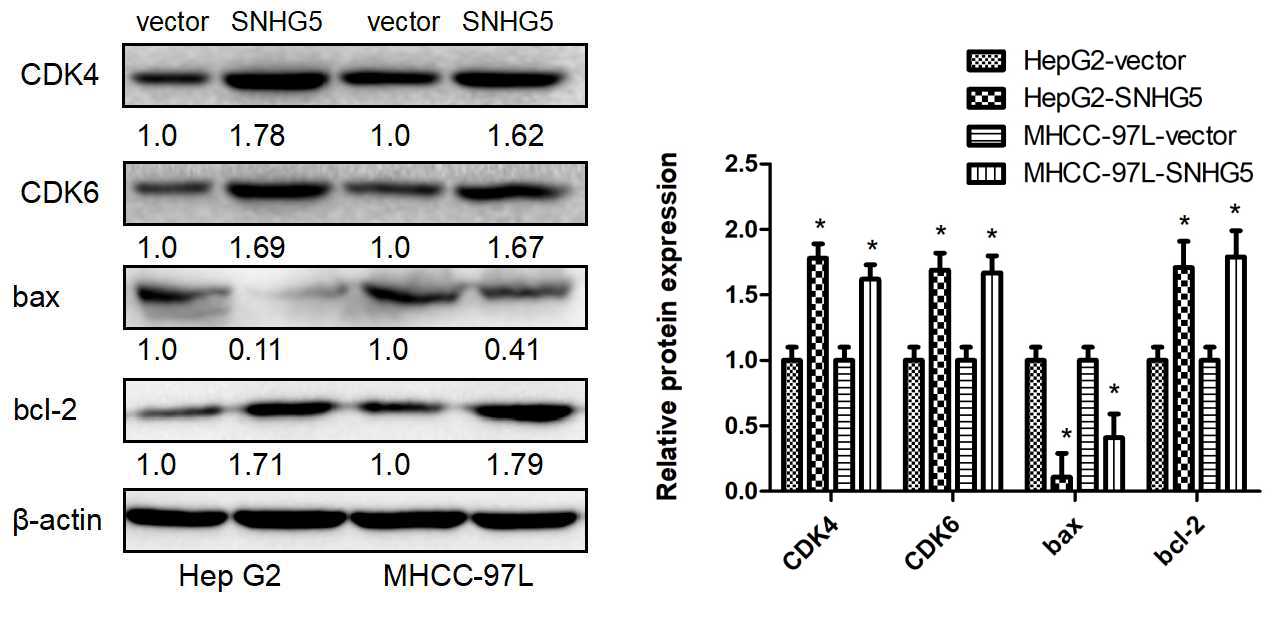
**

Fig.3G. Common cell cycle-related and apoptosis-related proteins expression levels detected by western blot analysis following SNHG5 upregulation. Quantitative analysis of protein relative expression by Image J.

**Figure3K**

**
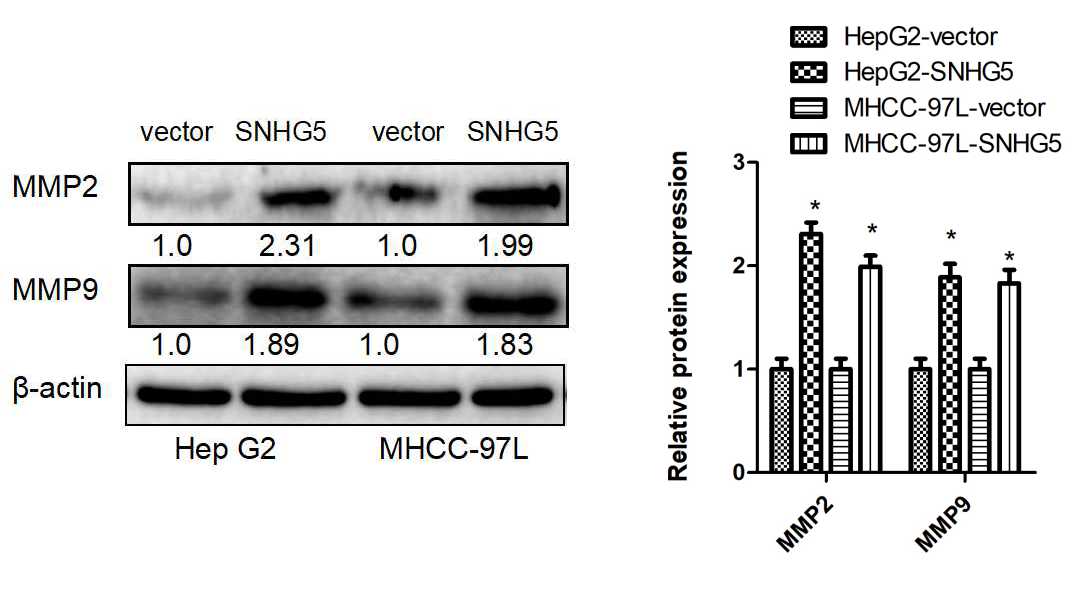
**

Fig.3K.The expression of MMP-2 and MMP-9 in HCC cells after SNHG5 overexpression were analyzed by western blot. Quantitative analysis of protein relative expression by Image J.

**Figure5G,I**

**
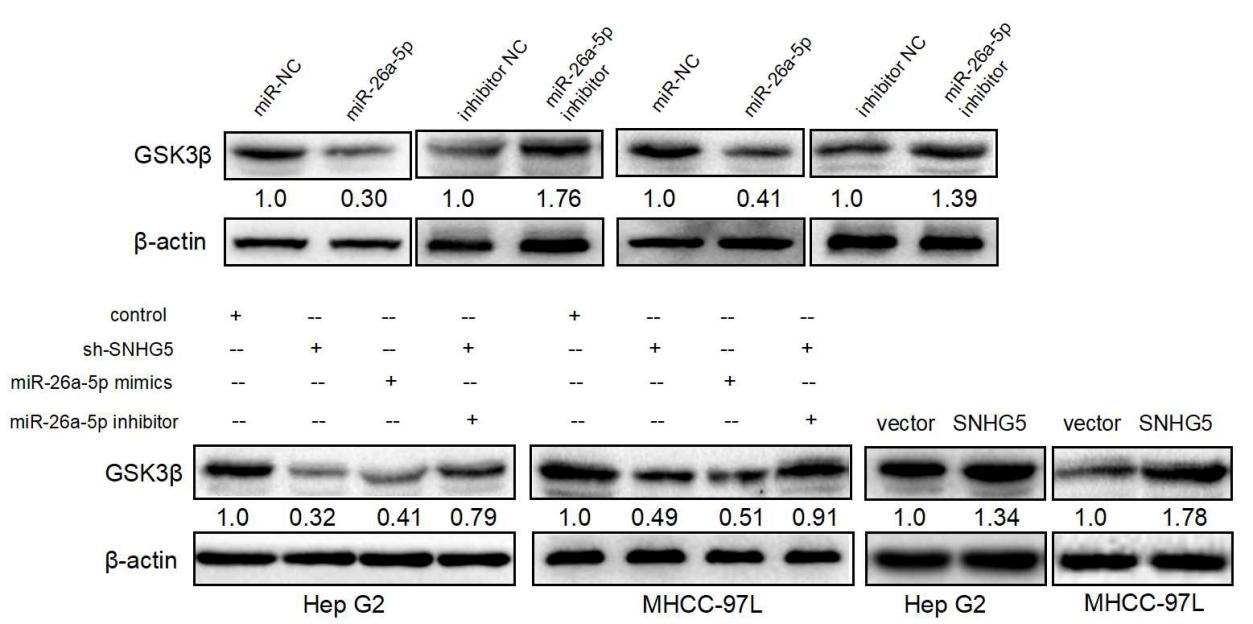
**

Fig.5G.The GSK3β protein levels after miR-26a-5p overexpression or downregulation were deceted by western blot. Fig.5I.Western blot analyses of GSK3β expression after knockdown or upregulated SNHG5, and the inhibition of miR-26a-5p co-tranfection.

**Figure7C**

**
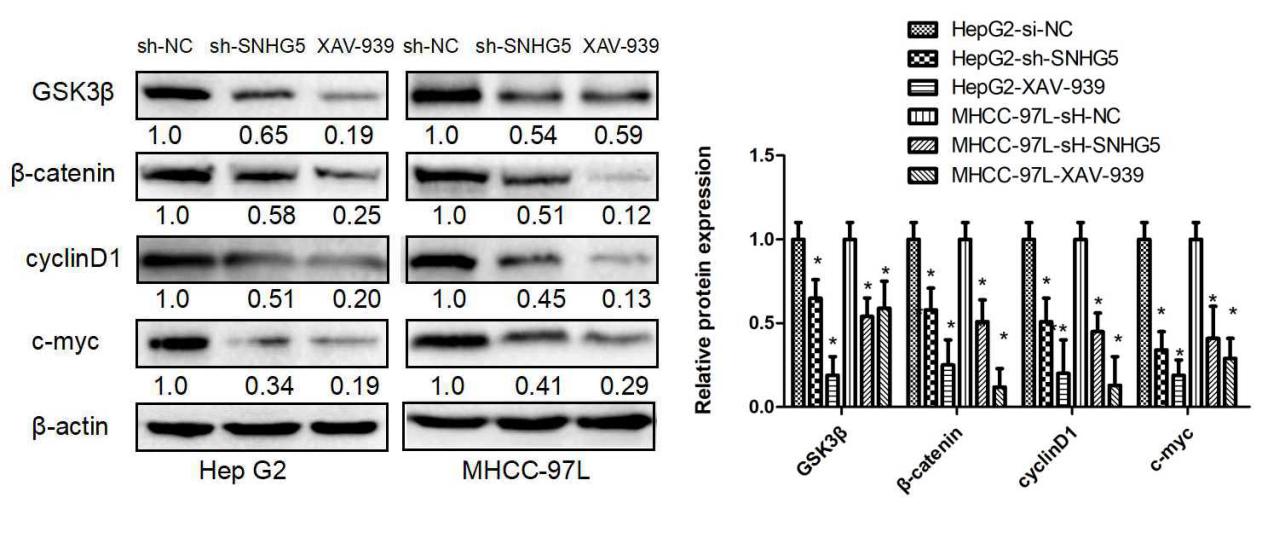
**

Fig.7C.Western blot analysis of the key components of Wnt/ β-catenin pathway protein expression following SNHG5 knockdown or co-transfection with shRNA and miR-26a-5p inhibitor. Quantitative analysis of protein relative expression by Image J.

**Figure7E**

**
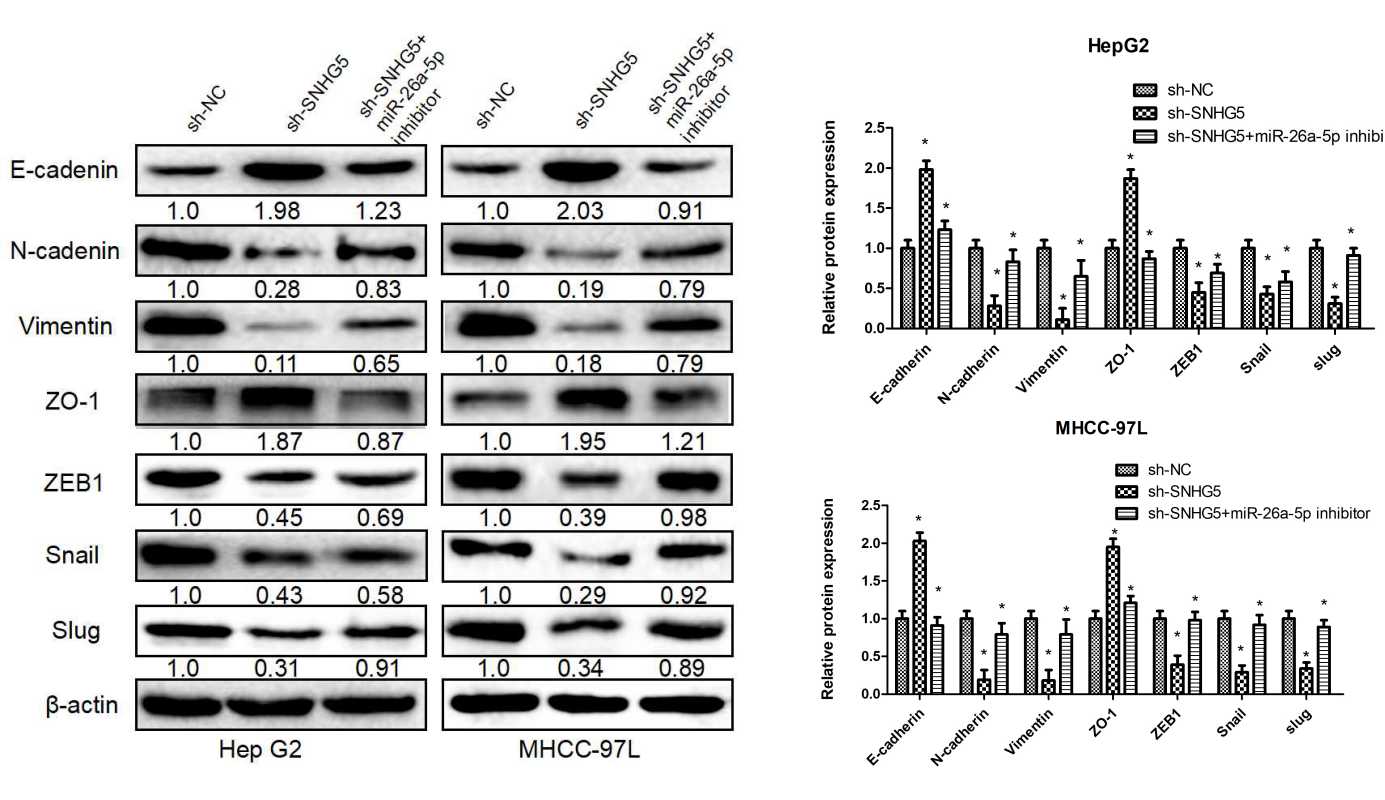
**

Fig.7E.Western blot analysis of the EMT markers in HCC cells after transfected with shRNA- SNHG5 or co-transfection with shRNA and miR-26a-5p inhibitor. Quantitative analysis of protein relative expression by Image J.
