# Supplementary material for: Long non-coding RNA SNHG5 promotes human hepatocellular carcinoma progression by regulating miR-26a-5p/GSK3β signal pathway
Source: Cell Death Dis. 2018 Aug 30;9(9):888. doi: 10.1038/s41419-018-0882-5 (PMC6117363; doi:10.1038/s41419-018-0882-5)
Supplement: Supplementary file 2 — Supplementary Tables [file 41419_2018_882_MOESM2_ESM.docx]

**Supplementary Tables**

**Supplementary Table 1. Primer sequences and target sequences used in this study**

| **Gene** | **Sequence or Target Sequence** |
| --- | --- |
| SNHG5-F | 5'-CGAGTAGCCAGTGAAGATAATG-3' |
| SNHG5-R | 5'-CACACAACAGTCAAGTAAACC-3' |
| β-actin-F | 5'-ATCGTGCGTGACATTAAGGAGAAG-3' |
| β-actin-R | 5'AGGAAGGAAGGCTGGAAGAGTG-3' |
| GSK-3β-F | 5'-GCACTGTGTAGCCGTCTG-3' |
| GSK-3β-R | 5'-GAGGAGGAATAAGGATGGTAGC-3' |
| E-cadherin-F | 5'-ATTCTGATTCTGCTGCTCTTG-3' |
| E-cadherin-R | 5'-AGTCCTGGTCCTCTTCTCC-3' |
| N-cadherin-F | 5'-CATCATCCTGCTTATCCTTGTG-3' |
| N-cadherin-R | 5'-CATAGTCCTGGTCTTCTTCTCC-3' |
| Vimentin-F | 5'-GCTGGAAGGCGAGGCGAGGAGAG-3' |
| Vimentin-R | 5'-CAACCGTCTTAATCAGAAGTGTC-3' |
| miR-26a-5p-F | 5'-TTCAAGTAATCCAGGATAGGCT-3' |
| miR-26a-5p-R | 5'-CGGTAAGAAGATGGAACCATAA-3' |
| U6-F | 5'-CGCTTCGGCAGCACATATAC-3' |
| U6-R | 5'-AAATATGGAACGCTTCACGA-3' |
| SNHG5-siRNA #1 | 5'-CAGUGAAGAUAAUGAAUGUTT-3' |
| SNHG5-siRNA #1 | 3'-ACAUUCAUUAUCUUCACUGTT-5' |
| SNHG5-siRNA #2 | 5'-GCAACGAUUUCUGGCUAGUTT-3' |
| SNHG5-siRNA #2 | 3'-ACUAGCCAGAAAUCGUUGCTT-5' |
| SNHG5-siRNA #3 | 5'-CUUGACUGUUGUGUGAAAATT-3' |
| SNHG5-siRNA #3 | 3'-UUUUCACACAACAGUCAAGTT-5' |
| siRNA-NC-F | 5'-UUCUCCGAACGUGUCACGUTT-3' |
| siRNA-NC-R | 3'-ACGUGACACGUUCGGAGAATT-5' |
| miR-26a-5p mimics-NC | 5'-UUCUCCGAACGUGUCACGUTT-3' |
| miR-26a-5p mimics | 5'-UUCAAGUAAUCCAGGAUAGGCU-3' |
|  | 3'-CCUAUCCUGGAUUACUUGAAUU-5' |
| miR-26a-5p-inhibitor | 5'-AGCCUAUCCUGGAUUACUUGAA-3' |
| miRNA inhibitor NC | 5'-CAGUACUUUUGUGUAGUACAA-3' |
| SNHG5-shRNA | 5'-CAGUGAAGAUAAUGAAUGUTT-3' |
| SNHG5-shRNA | 3'-ACAUUCAUUAUCUUCACUGTT-5' |

**Supplementary Table 2. Antibodies used in this study**

| **Antibody (Item No.)** | **Antibody dilution** | | | **Specificity** | **Company** |
| --- | --- | --- | --- | --- | --- |
|  | **WB** | **IHC** | **IF** |  |  |
| β-actin(13E5) | 1:1000 |  |  | Rabbit monoclonal | Cell Signaling Technology |
| Bax(sc-20067) | 1:500 |  |  | Mouse monoclonal | Santa Cruz Biotechnology |
| Bcl-2(sc-509) | 1:500 |  |  | Mouse monoclonal | Santa Cruz Biotechnology |
| CDK4(sc-166373) | 1:500 |  |  | Mouse monoclonal | Santa Cruz Biotechnology |
| CDK6(sc-7961) | 1:500 |  |  | Mouse monoclonal | Santa Cruz Biotechnology |
| Caspase-3(8G10) | 1:1000 |  |  | Rabbit monoclonal | Cell Signaling Technology |
| Cleaved Caspase-3(Asp175) | 1:500 |  |  | Rabbit polyclonal | Cell Signaling Technology |
| MMP-2(D8N9Y) | 1:500 |  |  | Rabbit monoclonal | Cell Signaling Technology |
| MMP-9(D6O3H) | 1:1000 |  |  | Rabbit monoclonal | Cell Signaling Technology |
| Ki-67(sc-23900) | 1:1000 | 1:100 |  | Mouse monoclonal | Santa Cruz Biotechnology |
| GSK-3β(D5C5Z) | 1:1000 |  |  | Rabbit monoclonal | Cell Signaling Technology |
| β-catenin(D10A8) | 1:1000 |  |  | Rabbit monoclonal | Cell Signaling Technology |
| CyclinD1(EPR2241 ) | 1:1000 |  |  | Rabbit monoclonal | Abcam |
| c-Myc(D3N8F) | 1:1000 |  |  | Rabbit monoclonal | Cell Signaling Technology |
| E-cadherin(24E10) | 1:1000 |  | 1:200 | Rabbit monoclonal | Cell Signaling Technology |
| N-cadherin(D4R1H) | 1:500 |  | 1:200 | Rabbit monoclonal | Cell Signaling Technology |
| Vimentin(D21H3) | 1:1000 |  | 1:100 | Rabbit monoclonal | Cell Signaling Technology |
| Snail (C15D3) | 1:1000 |  |  | Rabbit monoclonal | Cell Signaling Technology |
| Slug (C19G7) | 1:1000 |  |  | Rabbit monoclonal | Cell Signaling Technology |
| ZO-1(D7D12) | 1:1000 |  |  | Rabbit monoclonal | Cell Signaling Technology |
| ZEB1(D80D3) | 1:1000 |  |  | Rabbit monoclonal | Cell Signaling Technology |
